# Are the Teeth Deteriorating More Rapidly at the Present Time Than in the Past, and If so, Why?

**Published:** 1903-07-15

**Authors:** F. H. Essig


### ARE THE TEETH DETERIORATING MORE RAPIDLY AT THE PRESENT TIME THAN IN THE PAST, AND IF SO, WHY?

BY F. H. ESSIG, D.D.S.

Read before the Central Michigan Dental Association, May 14, 1903.

I hesitate in presenting so important a subject, fearing that I cannot do justice to it, but with your kind consideration I shall endeavor to bring out a few thoughts on the affirmative of the subject, hoping you shall profit by the discussion that may follow.

It is very difficult to give statistics upon this subject, and therefore I must depend upon personal observations in my own practice for the arguments which I shall give.

We are told by an eminent writer that in some excavations during 1901 in the sight of the old Gray Friar's Monastery, eleven skeletons of adult men were dug up, and in the skulls of all these skeletons of the fourteenth century the teeth were absolutely perfect; not a missing tooth nor a decay in any one of them.

I ask, can we do that in the twentieth century? No, instead of finding skulls with perfect teeth, we should find finely-constructed bridges, gold fillings and occasionally an amalgam filling. We are also liable to find plates of gold, aluminum and vulcanite.

I believe you will bear me out in the assertion that out of eleven skeletons of adult men taken from the graves of to-day scarcely one would have perfect teeth. What a pleasure it is to have a patient sit in your chair and upon examination you find the teeth sound in every respect. You gaze upon the patient and pronounce him a wonder.

The past month I have examined the mouths of twenty children, ages running from seven to ten, in order to gain some statistics upon this subject, and in every case I found decay. Many of them had all four first molars, with from one to two cavities in each, and many of the deciduous teeth badly decayed and broken down.

Another proof, that deterioration of the teeth is increasing faster to-day than ever before is the large number of dentists that are being graduated in this the twentieth century.

The demand has become so great the we are specializing along different lines such as Orthodontia, Crown and Bridge-work, Extracting, Porcelain Inlay and Prosthetic work. When you stop and think of the vast number of large laboratories that are being run in all of the larger cities turning out hundreds, yea thousands, of artificial dentures, in fact all classes of prosthetic work simply because the dentists have not the time to do it (as they claim), is not this certainly a good proof that our teeth are deteriorating faster to-day than ever? This was not the case fifteen years ago.

The first question is perhaps much easier answered than the last, but I hope I may give a few thoughts from my experience and observation that may help to enlighten us upon this important subject as to why the teeth deteriorate faster now than ever before, and the first is lack of education along this line, and secondly, carelessness.

In taking up the first of these propositions, I will ask whose fault it is that the public in general lacks this education? Is it the fault of our public school system, our colleges or our great universities? No, it is the fault of the dental profession. This is a broad assertion, but I believe I can prove it.

In the first place, nearly every day we are confronted with people of all classes trying to make us believe that their children's first molars, or first permanent teeth, are baby teeth and they stare at us with amazement, when we inform them that they are not, but permanent.

The dentist who is worthy of the name of his profession will stop right there and give a few minutes' lecture on the teeth to this patient, thus enlightening at least this one on this subject. This is not a common occurrence, but happens very often; at least I have found it so in my general practice.

Of course, our time is well spent in doing so. This is one way whereby we can enlighten a few of the many, but as a rule the information comes too late. The teeth are beyond our aid and in a majority of cases the little patients must suffer loss of this most important tooth, causing a mal-occlusion of the remaining teeth and leaving an impression that is undesirable upon the patient's mind as to the desirability of having any more dental operations performed. How can we, as dentists, overcome this objectionable feature in our pratice?

We should first do away with all petty jealousies, and get together and prepare proper literature upon this subject; sending it broadcast, not as Dr. Jno. Smith's idea, but from the general profession as a whole.

Secondly, let us work for dental education in our public schools by popular lectures and proper text-books, whereby the proper instruction can be instilled into the minds of the children, from the kindergarten to the graduating class. I believe this is a great deal more important than the teaching of French and German, or even Latin, to the children. Dr. Chas. R. Hambly, of Bradford, Pa., publishes several pamphlets or booklets such as "The American Dental Instructor," "Dental Bridge-work of To-day," and a few others that may be all right as far as the instruction goes, but this plan is selfish. His sole idea is to build up one's individual practice and not to better mankind in general. If the dentists of a community desire to use these booklets, let them use them collectively, thus avoiding the impression that Dr. Jno. Smith was the only dentist in town.

We are told that one of the principal causes of decay is poverty of the salivary secretion. The salivary glands are often weakened in early life by tubercular invasion, but apart from that there is, I conceive, a general reduction in their size and activity in civilized races. In the savages we find large jaw bones and powerful muscles of mastication, in which they can masticate their crude food and thus stimulate the secretion of saliva, giving them a copious supply of this fluid.

At the present time we do not find this state of affairs, but instead, our foods are softer and more pulpy, thus we permit our muscles to become less active and we find the salivary glands weakened.

You will all agree with me when I say that the salilva is the best cleanser and lubricant of the teeth, and I believe this is the reason why in some degree the teeth of the savages are better preserved. A copious supply of saliva assists digestion and any diminution in the quantity or deterioration of its quality must be injurious to the teeth.

The digestive power of the stomach to-day is not what it was in years past. We have but to look around us and see the increased demands for all kinds of peptonized foods, or foods that are partially digested before they are eaten so as to spare a weak stomach all possible exertion.

Dyspepsia is frequent and other diseases of the stomach are apparently more frequent than ever before.

The second and great cause of decay, to-day, is carelessness on the part of the parents. The majority of fathers and mothers pay little or no attention to their children's teeth until the little ones complain of pain. Truly such a state of affairs is unecessary and to be avoided.

What we must do, is to reach the homes and place proper literature therein and make the parents feel the responsibility that rests upon them. When we stop and note the amount of ignorance that exists concerning proper care of the teeth, and the little importance that the majority of parents attach to it, then it is no wonder that we have this condition.

I believe if the teeth could be closely watched and properly cared for, from the age of four to twenty years while the children are under their parents' care, we should see a great change for the better. In order to bring about a better condition in this respect, we, as a profession, must work hand in hand for the better education of the parents who have charge of the children at the critical periods in their lives. Proper habits of caring for the teeth will never be formed unless the parents are sufficiently intelligent to realize the importance of very early and persistent training of their little ones in the exercise of the principles of oral hygiene.

